# How capable is non-invasive EEG data of predicting the next movement? A mini review

**DOI:** 10.3389/fnhum.2013.00124

**Published:** 2013-04-08

**Authors:** Pouya Ahmadian, Stefano Cagnoni, Luca Ascari

**Affiliations:** ^1^Henesis s.r.lParma, Italy; ^2^Ibis Lab, Department of Information Engineering, University of ParmaParma, Italy

**Keywords:** non-invasive electroencephalography (EEG), brain computer interfaces (BCIs), single-trial analysis, event-related potentials (ERP), prediction of next movement, voluntary movements

## Abstract

In this study we summarize the features that characterize the pre-movements and pre-motor imageries (before imagining the movement) electroencephalography (EEG) data in humans from both Neuroscientists' and Engineers' point of view. We demonstrate what the brain status is before a voluntary movement and how it has been used in practical applications such as brain computer interfaces (BCIs). Usually, in BCI applications, the focus of study is on the after-movement or motor imagery potentials. However, this study shows that it is possible to develop BCIs based on the before-movement or motor imagery potentials such as the Bereitschaftspotential (BP). Using the pre-movement or pre-motor imagery potentials, we can correctly predict the onset of the upcoming movement, its direction and even the limb that is engaged in the performance. This information can help in designing a more efficient rehabilitation tool as well as BCIs with a shorter response time which appear more natural to the users.

## 1. Introduction

The concept of prediction in motor tasks was first introduced by Helmholtz in 1867 in an attempt to explain how humans localize objects. Later on, the concept of efference copies was published by Von Holst and Sperry in the fifties stating that motor commands create an internal copy which reflects the predicted movement and its resulting sensations (Blakemore et al., 1998; Wolpert and Flanagan, 2001). Since then, the idea that humans predict the consequences of their motor tasks has appeared as a prominent theory in all aspects of sensorimotor commands. Generally, prediction refers to estimating future states of a system.

Moreover, researches show great potential in the analysis of brain rhythms and event-related potentials (ERP) recorded by electroencephalography (EEG). Through EEG data acquisition, the current state of the brain can be understood in real time. As also mentioned above, part of this current state is a prediction of the next motor task. Therefore, by understanding and analyzing the brain rhythms and ERPs, the future motor commands can be predicted. This combination can lead to better rehabilitation tools for people with physical challenges. It might even have a potential to be used as supportive gadgets for healthy individuals.

In general, EEG recordings can be divided into two categories: non-invasive EEG recordings obtained from electrodes attached to the scalp surface and invasive EEG recorded from electrodes implanted inside the cranium. Since implanted electrodes in invasive EEG are closer to the brain than scalp electrodes, they can record the brain signals with higher amplitudes and smaller spatial scales ranging from a single neuron cell to distributed cell groups. Additionally, it is assumed that invasive EEG recording does not suffer from major artefacts that compromise non-invasive EEG, such as eye blinks. However, invasive EEG suffers from some technical difficulties and have significant clinical risks; because recording electrodes are implanted in the cortex and are required to function well for a long time, there is a risk of infections and other damage to the brain (Engel et al., 2005; Ball et al., 2009). Many studies have shown that although non-invasive EEG is less accurate in comparison with invasive EEG, it still contains enough real-time information to be used as a source for different applications and even in real-time brain computer interface (BCI) machines oriented to tasks such as word processing, Internet browsing or controlling a two-dimensional movement (Wolpaw and McFarland, 2004; Müller and Blankertz, 2006; Citi et al., 2008). Thus, in this article, the main focus is on non-invasive EEG single-trial analysis which from now on is shortened to EEG.

Another concept to address is the meaning of “before the movement” or “pre-movement” activity. This phenomenon refers to the time when no muscle movement is detectable or irrelevant if it occurs, but the subject is completely aware of the action that he is going to perform in the near future and it is also referred to as planning/preparation of the movements (Crammond and Kalaska, 2000; Toni and Passingham, 2003). In this time interval, which ranges from 500 ms to 2 s before movement onset, the cortex is preparing for action execution. As will be seen in section 2, there is a noticeable change in brain waves that only happens before movement.

This article aims to investigate the combination of the concepts of EEG and prediction, to see, based on today's knowledge and previous experiments, if it is possible to predict the next motor movement using current EEG recording. In the next section, we describe the main changes in the EEG data reported just before the voluntary movement. Then, in section 2 we see how the knowledge of these changes can be used to extract information about the upcoming movement. In each case, we address the EEG setup and protocol used and discuss the main foundation of the study. Finally, in the discussion and conclusion section we summarize the main ideas with the hope to draw more attention to the richness of pre-movement and pre-motor imagery EEG.

## 2. Changes in brain rhythm reported just before the movement

In this section, we address the changes detectable in EEG before the onset of the movement or motor imagery. All the following phenomena have been reported both when the movement is actually executed and when it is imagined as a part of the motor preparation procedure. One or a combination of these changes are the main focus of the studies trying to extract features from pre-movement or pre-motor imagery period discussed in the next section.

### 2.1. Bereitschaftspotential or readiness potential

Bereitschaftspotential (BP) or readiness potential (RP) is a negative cortical potential which starts to develop around 1.5 to 1 s before the onset of a voluntary movement and has two main components. The first component, also called “early BP,” is a slow-rising negative segment which begins about 1.5 s prior to the movement onset and is more prominent in the central-medial scalp. The other component, the “late BP,” has a steeper slope and occurs around 400 ms prior to the movement onset, having maximum amplitude over the primary motor cortex (M1). BP is an ERP, since its onset is time-locked to an event such as movement (Jahanshahi and Hallett, 2003; Shibasaki and Hallett, 2006). BP is proven to be evoked not only when a movement is performed by the subject, but also when the execution of an action by others is observed or even when the movement is imagined (Pineda et al., 2000; Kilner et al., 2004). Two of the most recent studies which focus on detection of BP are Ahmadian et al. (2012) and Ahmadian et al. (in press).

### 2.2. Alpha and beta event-related desynchronization

A short-lasting block/decrease of frequency power or event-related desynchronization (ERD) in the alpha band (about 8–12 Hz) and in the central beta band (about 16–24 Hz) has been reported beginning about 2 s before self-paced movement or motor imageries (Pfurtscheller and Neuper, 2003).

### 2.3. Contingent negative variation

Contingent negative variation (CNV) is a slow negative wave that develops in the interval between a “Warning” and a “Go” stimulus and shows anticipation for a forthcoming signal and preparation for execution of a response. In other words, CNV reflects preparation for *signaled* movements and is an index for *expectation*. The earlier segment of the CNV has maximum amplitude over the frontal cortex and is generated in response to a “Warning” cue. The later or terminal CNV (tCNV) begins around 1.5 s before the “Go” cue, it reflects preparation for motor response and has maximum amplitude over the motor cortex (M1) (Rohrbaugh et al., 1976; Brunia, 2003).

CNV potential has not been the main focus of the studies, although some studies have used a cue-based delayed protocol which can trigger this potential and thus affect the results reported (Hammon et al., 2008; Wang and Makeig, 2009; Lew et al., 2012). The one study that actually focused on detecting this potential states that it was not identified as the main pre-movement/pre-motor imagery feature in most of the subjects (Morash et al., 2008).

## 3. Studies

In this section we consider the studies that extract information from the pre-movement or pre-imageries EEG data. Some studies show the effectiveness of data acquired in this period in the real-time BCIs. Each study used a different EEG data acquisition including the electrodes montage and signal enhancement which is also discussed, since it is directly related to the results reported. We organized the studies in three groups based on the aims and findings of authors: predicting the onset of the next movement, the direction of next movement and also the limb engaged in the movement. These studies prove that with right EEG setting and signal processing, significant information can be extracted about the movement yet to come (see Table 1).

**Table 1:** **Studies on Pre-movement or Pre-imagery motor task categorized by the main findings**.

**References**	**Subjs #**	**EEG montage**	**Acquisition protocol**	**Brain feature(s)**	**Prominent preprocessing**	**Classifier**	**Key findings**
**PREDICTION OF ONSET OF MOVEMENT**							
Haw et al., 2006	5	1 Electrode between C3 and A1	Finger movement after visual cues	BP	Building specific BP template for each subject during 3 or 4 training session	Thresholding based on error and correlation	Detected the movement with an average accuracy of 70% and a low false positive rate
Bai et al., 2011	7	27 Electrodes	Hand wrist extension movement without cues	ERD	Spatial filter using Surface Laplacian derivation, bandpass filter (8–30 Hz) and electrodes reduced to 14	Mahalanobis linear distance	Prediction of movement with an average true positive rate of 75 ± 10% of total predictions about 0.62 s before the movement onset
Niazi et al., 2011	15	10 Electrodes focused on motor cortex	Ankle dorsiflexion movement without cues	BP	Bandpass filter (0.05–10 Hz), extracted a template from the training data set using spatial filter and sliding a window of 2 s wide with 200 ms shifts	Neyman Pearson lemma	Predicted the movement with an average true positive rate of 82.5% around 187 ms before movement onset
Lew et al., 2012	12	64 Electrodes	Hand movement at least 2 s after sound cues	BP	Bandpass filter 0.1–1 Hz, electrodes reduced to 6 placed over the central motor cortex and sliding a window of 500 ms wide with 10 ms shifts from 2500 ms before movement onset to 1000 ms after	Linear Discriminant analysis	Predicted the movement with maximum average true positive rate of 81% around 140 ms before movement onset
**PREDICTION OF DIRECTION OF THE MOVEMENT**							
Lakany and Conway, 2007	4	28 Electrodes	Move the manipulandum placed in their hands toward the direction cue	BP and ERD/ERS	Artefact removal, low-pass filtering at 50 Hz, electrode reduction to 1 (*C*3) and extracting spatio-temporal features via CWT	Wrapper based on SVM	Average accuracy of 81.5% on the test dataset for two different directions
Hammon et al., 2008	2	64 Electrodes	Delayed reaching and touching of screen corner pointed by a directional cue	Not stated explicitly: BP, ERD, CNV	Artefact rejection, first 500 ms of the delay period used for analysis and extracting 8 vectors of different features	Multinomial logistic regression classifier	Distinguishing left from right targets is more effective than discriminating top from bottom targets
Wang and Makeig, 2009	4	128 Electrodes	Delayed movement protocol; gazing toward the direction cue, reaching for it with one hand, or both activities	Not stated explicitly: BP, ERD, CNV	Segmented 700 ms after direction cue, baseline correction, removal of noisy electrodes and spatial filtering with ICA using Extended Informax algorithm	SVM classifier using an RBF kernel	Pre-movement EEG signals carry information about the direction of the intended movement, classification of go-left and go-right planning with average accuracy of 80.25 ± 2.22
**PREDICTION OF TYPE OF THE MOVEMENT**							
Blankertz et al., 2001	1	27 Electrodes	Pressing the computer keyboards with fingers of both hands at an average speed of 1 key every 2 s	BP	Artefact rejection, low-pass filtering at 5 Hz, sub-sampling at 20 Hz and electrodes reduced to 21	Learning machines, e.g., SVM	Discrimination between left and right-hand finger pre-movement on average 100–230 ms before key pressed with 96% classification accuracy
Blankertz et al., 2003	8	32, 64, or 128 Electrodes	Self-paced pressing one of four keys, using the fingers of the right or left-hand	BP	bandpass with Fourier transform between 0.4 and 3.5 Hz, sub-sampling at 20 Hz and electrodes reduced to 23 over motor cortex	Classifier based on Fisher's Discriminant	Discrimination between left and right-hand finger pre-movement as early as 120 ms before the movement onset and as fast as 2 taps per second
Morash et al., 2008	8	29 Electrodes over sensorimotor areas	Delayed protocol; to perform or imagine right-hand, left-hand, tongue, or right-foot move after a “Go” cue	CNV and ERD/ERSs	Artefact rejection, spatial filtering via ICA and temporal filtering via DWT	Naive Bayesian classifier	Predicting which of the four movements/imageries is about to occur is possible and it is manifested stronger in the ERD/ERS in comparison with CNV

*“Subjs #” indicates the number of subjects who participated in the experiment*.

### 3.1. Predicting the intention of movement

Studies carried out in Haw et al. (2006); Bai et al. (2011); Niazi et al. (2011) and Lew et al. (2012) try to answer the question whether or not the subject wants to move in the short future. They do not try to determine the kind or the direction of movement but to detect the upcoming of any movement. In these studies, the movement onset is not used as a prior in the detection method in order to simulate the full meaning of prediction.

In Haw et al. (2006) the authors implemented a user-specific template matching structure as part of a method to detect movement planning. Here, the focus was more to detect the movement rather than to predict it. A BP waveform recorded via only one electrode was used to build the template. The results showed detection of movement onset with an average accuracy of 70% and a low false positive rate.

Authors in Bai et al. (2011) focused on prediction. In their experiment, subjects were asked to perform three sessions in each of which the only task was to perform a right-hand wrist extension movement whenever they wanted, without any cue (self-paced movement). EEG data from the first two sessions were used for training and applied to the third session to test the predictions. The work was based on detection of the ERD or power decrease in alpha and beta bands in single trials. The study reported an average true positive rate of 75 ± 10% of total predictions, about 0.62 s on average before the movement onset.

In Niazi et al. (2011), the authors detected the onset of the movement as early as 187 ms before the movement with an average accuracy of 82.5%. The subjects were asked to perform a self-paced ankle dorsiflexion. Each subject performed 5 runs: 2 were used for training and the rest for the testing phase. The detection method was based on movement-related cortical potentials such as BP; a template was extracted from the training data sets using a spatial filter. The detection decision was based on the correlation computed between spatially filtered channels of the test data sets and the template in a sliding window.

In Lew et al. (2012), the authors showed that it is possible to predict the movement 500 ms prior to its occurrence. Here, the subjects were asked to move their hands at least 2 s after hearing an auditory cue. In the training phase, the signal preceding the movement by 500 ms was used in comparison with 500 ms before the auditory cue. For the testing phase, a shifting window was implemented. Their results showed maximum average true-positive rate of 81% peaking 140 ms before movement onset across subjects.

### 3.2. Predicting the direction of the movement

In studies done by some researchers (Lakany and Conway, 2007; Hammon et al., 2008; Wang and Makeig, 2009) it was shown that it is possible to determine the direction of the moving limb prior to movement onset. In Lakany and Conway (2007), the authors analyzed 3 s of data before movement onset. The subjects were asked to move a manipulandum placed in their hands toward the direction indicated by a cue on the screen. For EEG analysis, spatio-temporal features were extracted via continuous wavelet transform (CWT). Then, they performed a wrapper method based on support vector machines (SVM) for feature selection and classification and reported an average accuracy of 81.5% during testing for two different directions.

In the study reported in Hammon et al. (2008), the subjects were asked to reach and touch the corner of the screen indicated by a directional cue. The delay between the direction cue and go cue was randomized between 750 and 1500 ms. Only the EEG data of the first 500 ms of the delay period were analyzed by three classifiers: a four-class classifier for reaching the target, and two binary classifiers of left vs. right reaches and top vs. bottom reaches. The results of the binary classifiers showed that distinguishing left from right targets is more effective than discriminating top from bottom targets.

**Figure 1:**
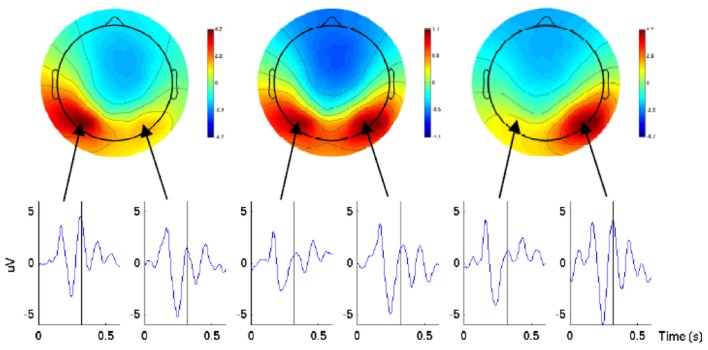
**Scalp maps and ERP waveforms of the back-projected parietal ICs for one subject in three conditions (left, center, and right) 320 ms after the direction cue.** The ERP waveforms were from the two lateral parietal electrodes with the highest amplitude projection of cortex. The picture is taken from Wang and Makeig (2009).

In a follow-up work in Wang and Makeig (2009) the authors showed that EEG signals obtained from the posterior parietal cortex before the movement onset carry information about the direction of the intended movement. A delayed movement protocol was designed where the movement consisted of tracking the target with gaze, reaching for it with one hand without eye movement, or doing both actions. It had to be performed 700 ms after the subject was made aware of the task direction by a cue; this was the period which was then used for data analysis. For EEG analysis, they used independent component analysis (ICA) performed by the Extended Infomax algorithm. Two lateralized temporal-parietal components were identified in each subject's EEG data time locked to the onset of the movement cue. For a better understanding of direction coding, the parietal ICs were back-projected onto the scalp and visualized. The result produced a clear contralateral negativity and positivity with respect to the intended movement direction (see Figure 1). Moreover, using SVM, the authors obtained an average accuracy of 80.25% for single-trial classification of right vs. left.

### 3.3. Predicting the type of the movement

In this section, we consider the studies which try to determine the type of the movement or the body part that will move in a short future. In the previous section the movement was carried out by one limb and the direction of the movement was in question. However, some studies go beyond that to see whether it is possible to predict the future moving limb. One of the earliest work in this area is the work reported in Blankertz et al. (2001). In this work, using the BP features, authors managed to discriminate between left and right-hand finger pre-movement in keyboard typing on average 100–230 ms before the key was pressed. The actual time at which the key was pressed was determined by electromyography (EMG). The subject task was to press the computer keyboards with the index or little fingers of both hands in a self-paced manner, resulting in an average speed of 1 key every 2.1 s. Based on this research, BCI competition 2003, data set IV, self-paced tapping, was released (Blankertz, nd). Using one subject executing the same task, 416 epochs of 500 ms EEG were provided, each ending 130 ms before an actual key press. The epochs were randomly shuffled and split into 316 labeled epochs for the training set and 100 unlabeled epochs for the test set. Obviously, the goal of the competition was to submit the estimated labels for the test set with the smallest number of misclassifications. Needless to say, this data set attracted a lot of researchers to find a solution for the problem of predicting the next moving limb. A total of 15 groups submitted their results for this data set, 4 of which had a performance close to chance level (the error rate was higher than 43%) (Blankertz et al., 2004). The best submission was by Zhang and colleagues (Zhang et al., 2004) with an error rate of 16%. The use of this data set did not stop after the end of the competition and the release of the results; since this dataset is still available for download, it has become a benchmark and has been used by other research groups such as (Congedo et al., 2006).

In Blankertz et al. (2003), the authors used the concept of bit transfer to evaluate the speed and accuracy of their method in discriminating between left and right-hand figure movements in keyboard typing. The subjects were asked to press one of the four keys, using the fingers of the right or left hand, in a self-chosen order and timing. For data analysis, after preprocessing the data, a classifier based on Fisher's Discriminant was applied to the mean and covariance matrices calculated from the training data. Their results showed that not only could the discrimination between different limb movements be detected as early as 120 ms before their onset but that this was also achieved in motor sequences as fast as 2 taps per second.

In another study reported in Morash et al. (2008), the authors used the EEG signals preceding movement and motor imagery to predict which of the movements/motor imageries of different body parts (right hand, left hand, tongue, and right foot) was about to occur. The subjects were informed about the type of the movement by a cue and performed it at least 2 s after being informed by another “Go” cue. The time window of 1.5 s before the “Go” cue was used in the analysis of data which correspond to ERD/ERS and CNV oscillation. The results showed that the preceding movement and motor imagery ERD/ERS can be used to predict which of the four movements/imageries is about to occur. Prediction accuracy depended on signal quality. However, the highest average testing accuracies reported for the two-movement/imagery categories were related to right-foot and left-hand responses. The study also tried to identify CNV and ERD/ERSs on motor areas in single trials and concluded that, compared to CNV, the ERD/ERS is the most specific pre-movement/pre-motor imagery signal with respect to the movement/imagery that is about to be performed.

## 4. Discussion

In the studies above there is a close link between one or more brain rhythms and ERPs and the preprocessing step. In particular, the choice of the filter and of the placement of the electrodes depends on the brain features on which the method is based. For instance, (Blankertz et al., 2003) and (Lew et al., 2012), which were based on BP, used a bandpass filter with upper cutoff frequency of less than 4 Hz and on electrodes attached to the motor cortex. Consequently, more studies to further investigate the pre-movement period can improve the BCIs and, thus, the prediction of the next motor task.

This article is not a completely exhaustive review of the capability of EEG in predicting the next motor task. It is mainly aimed at providing examples of the progress in the field as well as stimulating ideas for new research proposals.

## 5. Conclusion and future work

In conclusion, the EEG data gathered before the forthcoming movement which corresponds to motor preparation and planning period of the brain show significant prediction potentials. The evidence from both Neuroscience and Engineering research support this hypothesis, even though this field has not been vastly explored. Further studies in both fields in this short pre-movement time can extend exploration of the frontiers of motor preparation and decrease the response time of BCIs. To know beforehand whether the subject is willing to move, in what direction, or using which limb can make it possible to determine the next course of actions in rehabilitation procedures more effectively. It can also decrease the response time of BCIs and let them appear more natural to their users. In some applications, such as driving, it may even have a more crucial role. Study reported in Haufe et al. (2011) showed that it is possible to predict an upcoming emergency brake up to 130 ms earlier than pedal response. At driving speed of $100\frac{\text{km}}{\text{h}}$, this time reduces the braking distance by 3.66 m, which can be very effective in terms of survival. They also showed that levels of predictive accuracy using EEG worked faster than EMG, suggesting the superiority of using EEG in such a context.

### Conflict of interest statement

The authors declare that the research was conducted in the absence of any commercial or financial relationships that could be construed as a potential conflict of interest.

## Funding

Pouya Ahmadian is funded by the European Commission (Marie Curie ITN MIBISOC, FP7 PEOPLE-ITN-2008, GA n. 238819).
